# The Sides of the Body

**Published:** 1892-10

**Authors:** 


### BOOK NOTICES.

THE SIDES OF THE BODY AND KINDRED REMEDIES, by Dr. C. von Bœnninghausen. Translated for THE HOMŒOPATHIC PHYSICIAN and issued as a supplement to Volume XII, 1892, by Dr. J. D. Tyrrell, Toronto, Canada. Philadelphia: THE HOMŒOPATHIC PHYSICIAN, 1125 Spruce Street, 1892. Twenty-seven pages, paper cover. Price, fifty cents.

Bœnninghausen's *Sides of the Body* is one of the most celebrated, as it is one of the most valuable of his works. It has been long out of print and has been therefore inaccessible to students of Homœopathy. Through the enterprise and industry of Dr. Tyrrell it is now reproduced with certain additions which will much enhance its value. These additions are readily discernible by being inclosed in brackets. Every earnest homœopathic physician should have it to assist him in the selection of the remedy. Like all works written by Bœnninghausen, it is to be used only as an aid in the study of the remedy, and is in no sense a substitute for the study of the materia medica.

